# Transcriptome Profiling Insights the Feature of Sex Reversal Induced by High Temperature in Tongue Sole *Cynoglossus semilaevis*

**DOI:** 10.3389/fgene.2019.00522

**Published:** 2019-05-29

**Authors:** Jinxiang Liu, Xiaobing Liu, Chaofan Jin, Xinxin Du, Yan He, Quanqi Zhang

**Affiliations:** ^1^Key Laboratory of Marine Genetics and Breeding, Ministry of Education, Ocean University of China, Qingdao, China; ^2^Laboratory for Marine Fisheries Science and Food Production Processes, Qingdao National Laboratory for Marine Science and Technology, Qingdao, China

**Keywords:** high-temperature treatment, RNA-seq, *cyp19a1a*, DNA methylation, *Cynoglossus semilaevis*

## Abstract

Sex reversal induced by temperature change is a common feature in fish. Usually, the sex ratio shift occurs when temperature deviates too much from normal during embryogenesis or sex differentiation stages. Despite decades of work, the mechanism of how temperature functions during early development and sex reversal remains mysterious. In this study, we used Chinese tongue sole as a model to identify features from gonad transcriptomic and epigenetic mechanisms involved in temperature induced masculinization. Some of genetic females reversed to pseudomales after high temperature treatment which caused the sex ratio imbalance. RNA-seq data showed that the expression profiles of females and males were significantly different, and set of genes showed sexually dimorphic expression. The general transcriptomic feature of pesudomales was similar with males, but the genes involved in spermatogenesis and energy metabolism were differentially expressed. In gonads, the methylation level of *cyp19a1a* promoter was higher in females than in males and pseudomales. Furthermore, high-temperature treatment increased the *cyp19a1a* promoter methylation levels of females. We observed a significant negative correlation between methylation levels and expression of *cyp19ala*. *In vitro* study showed that CpG within the cAMP response element (CRE) of the *cyp19a1a* promoter was hypermethylated, and DNA methylation decreased the basal and forskolin-induced activities of *cyp19a1a* promoter. These results suggested that epigenetic change, i.e., DNA methylation, which regulate the expression of *cyp19a1a* might be the mechanism for the temperature induced masculinization in tongue sole. It may be a common mechanism in teleost that can be induced sex reversal by temperature.

## Introduction

The types of sex determination are diversified in teleost. Three main types of primary sex determination have been described in gonochoristic species: genotypic sex determination (GSD), temperature-dependent sex determination (TSD) and a combination of both (GSD ++ TSD) (Ospina-Álvarez and Piferrer, 2008; Yamamoto et al., 2014). The sex of fish had strong uncertainty in the development process. In addition to genetic information, environmental factors could influence the sex determination, such as temperature. Apart from fish, the temperature irreversibly determining gonadal sex has been well established in reptiles and amphibians (Sarre et al., 2011; Flament, 2016). Since firstly described in *Menidia menidia* (Conover and Kynard, 1981), this phenomenon had been widely observed in fish, which showed that sex ratio would become unbalanced if the fish experienced high temperature during thermosensitive period (TSP). The imbalance of sex ratio was caused by sex reversal. Usually, it can be divided into three types: (1) high temperature has positive correlation with the proportion of males. (2) High temperature induces females, and has a negative correlation with males. (3) Both low temperature and high temperature increase the proportion of males (Baroiller and D’Cotta, 2001; Devlin and Nagahama, 2002; Ospina-Álvarez and Piferrer, 2008).

To verify the molecular mechanism of temperature effects (TE), a series of exploration was carried out. Steroid hormone, glucocorticoid, and epigenetic modification have been reported to be related to sex reversal and played critical roles during sex differentiation in TSD (Hattori et al., 2009; Lance, 2009; Nakamura, 2010; Yoshinaga et al., 2010; Navarro-Martín et al., 2011; Fernandino et al., 2012, 2013; Kitano et al., 2012; Piferrer, 2013; Zhang et al., 2013). Besides, it was discovered that intron retention of *JARID2* and *JMJD3* genes in *Pogona vitticeps* could mediate sex-reversed females (Deveson et al., 2017). Androgen-to-estrogen ratio determined whether an undifferentiated gonad differentiated into a testis or ovary in non-mammalian vertebrates (Simpson et al., 1994). The regulation of steroid ratio depended on the activity of gonadal aromatase, the product of *cyp19a1a*, which converts androgens into estrogens irreversibly (Simpson et al., 1994). In reptiles, up-regulating or down-regulating *cyp19a1a* could alter gonad phenotype. The expression level of gonadal *cyp19a1a* was associated with TSD in *Trachemys scripta* and *Alligator mississippiensis* (Pieau and Dorizzi, 2004; Matsumoto et al., 2016). In teleost, it has been confirmed that high temperature induced masculinization is related to the methylation level of *cyp19a1a* promoter in *Dicentrarchus labrax*. Methylation modification in the promoter region could suppress the binding of transcription factors to the corresponding sites (SF-1, FOXL2, and CREB) resulting in the change of expression (Navarro-Martín et al., 2011; Zhang et al., 2013). Similar conclusions were observed in *Oreochromis niloticus* and *Oncorhynchus mykiss* (Valdivia et al., 2014; Wang et al., 2017). Meanwhile, *FOXL2* and *SOX9*, which showed dimorphic DNA methylation patterning were also considered as the candidate genes in *A. mississippiensis* and *Paralichthys olivaceus* (Parrott et al., 2014; Si et al., 2016). Other factors have also been suggested to play a role in GSD + TE, such as heat shock proteins (*HSPs*), transient receptor potential channels (*TRPs*), cold inducible RNA binding proteins (*CIRBPs*), and microRNAs (Kohno et al., 2010; Rhen and Schroeder, 2010; Bizuayehu et al., 2015; Czerwinski et al., 2016; Schroeder et al., 2016).

The effect of temperature on the sex differentiation can be profound and far-reaching, and needs comprehensive studies to fully understand the molecular mechanisms. Chinese tongue sole, *Cynoglossus semilaevis*, is a GSD + TSD sex determination teleost with ZZ/ZW sex chromosomes (Zhou et al., 2005). Female-specific DNA sequences had been identified in *C. semilaevis*, which could be used for distinguishing genetic female and male (Wang et al., 2009, 2013). Therefore, *C. semilaevis* is a unique powerful model to explore molecular events associated with GSD + TSD. In previous study, it was reported that epigenetic modification was involved in sex reversal of *C. semilaevis* by BS-seq and RNA-seq, and transgenerational epigenetic inheritance was observed in offspring generated by sex reversal individuals (Chen et al., 2014; Shao et al., 2014). We aimed to filter genes related to sex differentiation, explore the relationship of expression level and methylation modification, and analyze whether methylation could regulate the binding of transcription factor. In this study, the genetic female individuals that inversed to phenotypic male individuals are defined as pseudomales. These pseudomales are distinguished from high temperature treatment groups using female-specific markers. RNA-seq was performed on the gonads of females, males, and pseudomales. The whole expression profiles were investigated, and candidate genes involved in sexual gonad development were identified. The methylation patterns of the putative genes were also analyzed. The interaction of upstream regulatory sequence and the corresponding transcription factors was verified by dual-luciferase reporter system. These findings helped us to understand the genetic epigenetic programing driving vertebrate GSD + TE and provide insight for future investigations aimed at clarifying the mechanisms controlling sex differentiation and sex reversal.

## Materials and Methods

### Fish Rearing and Temperature Treatment

**FIGURE 1:**
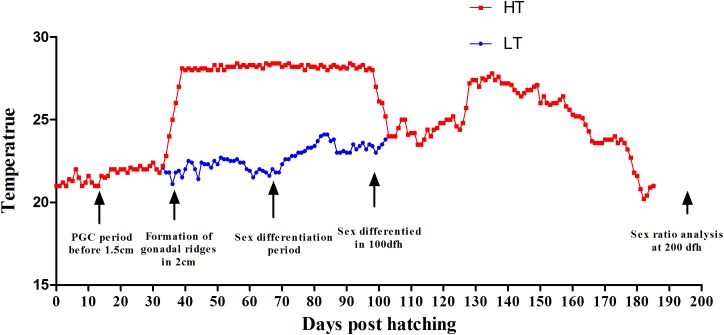
Thermal protocols applied in the present study. The experimental groups were: control group, LT, 20°C from 25 to 100 dpf, thereafter following the natural fluctuation (blue line). High-temperature group, HT, 28°C from 25 to 100 dpf, throughout the whole TSP (red line). The major events related to gonad formation and sex differentiation are also indicated.

Fish and embryos were collected from Yellow Sea Aquatic Product Co. Ltd., Shandong, China. Embryos were incubated at 20°C, the natural temperature for *C. semilaevis* spawning, fertilization and hatching. For this study, a batch of embryos collected from three pairs of parents was used. After hatching the fry were reared at ambient temperature (20–22°C). The juveniles at 25 days post fertilization (dpf) with total length (TL) of 13 ± 2 mm were separated into two groups. One group (*n* = 3000) was reared at ambient temperature throughout the TSP as control group (low temperature group, LT). The other group (*n* = 3000) was exposed at 28°C during the entire TSP and as the high-temperature group (high temperature group, HT). The temperature was increased to 28°C at a rate of 0.5°C/day, and then maintained for 100 days, until 125 dpf (Figure 1). Then the water was recovered to ambient temperature to follow the natural fluctuations until the end of the study, when the fish were 300 days old. The proportion of phenotypic males and females was counted by gonad biopsy and section confirmation in LT group and HT group, respectively. From these phenotypic males genetic males and pseudomales were identified using female-specific markers (Wang et al., 2009). The survival rate was also calculated for both of the groups.

### Sample Collection and Gonadal Histology

At 300 dpf, fish were sacrificed and gonadal samples were collected. For each fish, one gonad was processed for histological identification of phenotypic gender and DNA/RNA extraction. Gonads were fixed in 4% PFA in PBS, embedded in paraffin, cut at 7 μm thickness and stained with haematoxylin-eosin. Meanwhile, the other gonad was snap-frozen in liquid nitrogen and stored at -80°C for RNA-seq analysis. Muscle tissues were collected to extract DNA for individual sexing and methylation analysis. The methylation level of muscle was selected as control.

### RNA Isolation, cDNA Library Construction, and Illumina Sequencing

Gonads from nine individuals including three biological replicates of females (FO), males (MT), and pseudomales (PMT) were selected for RNA-seq analysis. Total RNA was extracted using Trizol Reagent (Invitrogen, Carlsbad, CA, United States) according to the manufacturer’s protocol, treated with RNase-free DNase I (TaKaRa, Dalian, China) to degrade genomic DNA, and then frozen at -80°C. RNAclean Kit was applied to remove proteins. The quality and quantity were evaluated via 1.5% agarose gel electrophoresis and spectrophotometry using NanoPhotometer Pearl (Implen GmbH, Munich, Germany) and Agilent 2100 Bioanalyzer (Agilent Technologies, Santa Clara, CA, United States).

The nine RNA-seq libraries were constructed with Illumina TruSeq RNA Sample Prep Kit (Illumina, San Diego, CA, United States) in accordance with the manufacturer’s instruction. Then the libraries were subjected to paired-end sequencing of 125 bp on the Illumina HiSeq 2000.

### Data Processing and Bioinformatics Analysis

Raw reads generated from the Illumina sequencing platform were cleaned by removing adaptors and low quality sequences using FastQC. The cleaned reads of each sample were mapped to the reference genome (Chen et al., 2014) by TopHat with default parameters (Kim and Salzberg, 2011). Then the mapping files were analyzed using Cufflinks to assemble the reads into transcripts for each dataset (Roberts et al., 2011). Complete transcripts were obtained by merging the assemblies of all datasets using Cuffmerge.

### Identification of Differentially Expressed Genes and Functional Enrichment Analysis

All the expressed genes were aligned to databases for homology annotation, including non-redundant protein databases (NR), Swiss-Prot, Gene Ontology (GO), eukaryotic Orthologs Groups (KOG), and Kyoto Encyclopedia of Genes and Genomes (KEGG) by BlastX with *e*-value of 1e-5 (Kanehisa et al., 2008).

FPKM were used to select the DEGs. The FPKM was calculated by Cuffdiff (Trapnell et al., 2012). To identify the differentially expressed genes (DEGs) among female, male and pseudomale gonads, we set the following standards: genes with an adjusted log2FoldChange ≥ 2 or log2FoldChange ≤-2, and *P* < 0.01 were considered as DEGs. The DEGs were then enriched by GO terms and KEGG categories using DAVID (Huang et al., 2008). The visualization of global similarities and differences of expression profiles of all individuals was accomplished by principle component analysis (PCA), MA plot and heatmap. These analysis were completed with R package.

### qRT-PCR Validation

A total of ten DEGs (*Sox9, GATA4, Dmrt1, AMH, HSD11b2, cyp19a1a, esr1, topaz1, GATA6, Sox3*) were selected for qRT-PCR validation. Specific primer pairs were designed by IDT. qRT-PCR was performed in a 20 μg solution containing 10 ng of template cDNA and SYBR qPCR SuperMix (Novoprotein, Shanghai, China) by using LightCycler 480 (Roche, Forrentrasse, Switzerland) at 95°C for 5 min pre-incubation, followed by 45 cycles of 95°C for 15 s and 60°C for 45 s. The relative quantities of the target genes expressed as fold variation over *GAPDH* were calculated using the 2^-ΔΔCt^ comparative Ct method. qRT-PCR data were statistically analyzed using one-way ANOVA followed by LSD test using SPSS 20.0. *P* < 0.05 indicated statistical significance.

### Methylation Levels Measured by Bisulfite-Mediated Genomic Sequencing

Methylation sites were prediction and BSP primers design in promoter were performed by Methprimer. Gonad and muscle tissues of females, males and pesudomales (six individuals each) were used to extract genomic DNA. The DNA samples from the same tissue of the same gender were mixed. The mixed DNA was modified using the EZ DNA Methylation-Gold Kit (ZYMO Research). The primers M-*cyp19a1a*-Fw1/Rv1 and M-*cyp19a1a*-Fw2/Rv2 were used for methylation-specific PCR. Eight positive clones were sequenced for each group. Site-specific methylation measurements were analyzed using BiQ-Analyzer.

### *Cyp19a1a*-Luc Reporter Vector Construct and *in vitro* Methylation

A pGL3-*Cyp19a1a*-Luc reporter vector was constructed by inserting the *cyp19ala* promoter fragment into the pGL3-basic vector (Promega, Madison, WI, United States) between *Sac*I and *Xho*I sites. The promoter was a 1969 bp fragment amplified from genomic DNA with primers pGL3-*cyp19a1a*-Fw/Rv (Supplementary Table S3). The pGL3-*cyp19a1a* promoter vector was cytosine-methylated using M. SssI methylase (Thermo Fisher Scientific, MA, United States) (M-*cyp19a1a*-Luc) according to the manufacturer’s instructions. It could methylate all cytosine residues within the double-stranded dinucleotide recognition sequence. The methylation status of the vector was checked by *Hha*I, which only digested methylated DNA.

### Transfection and Luciferase Reporter Gene Assay

The HEK 293T cell line was used for transfection with unmethylated and methylated plasmids. Before the experiment, a total of 5 × 10^5^ cells were seeded into 24-well plates and cultured for 24 h. Then the plasmids were transfected into HEK 293T cells by Lipofectamine^TM^ 3000 Transfection Reagent (Thermo Fisher Scientific, MA, United States) according to the manufacturer’s instructions. At 48 h after transfection, cells were washed with PBS and analyzed for Luc activity using the luciferase assay system (Promega, Madison, WI, United States). Forskolin (5 μM), the activation of cAMP, which binds to CREB site, was added 10 h before the end of cell culture.

## Results

### Sexual Ratio Changes After High Temperature Treatment

**FIGURE 2:**
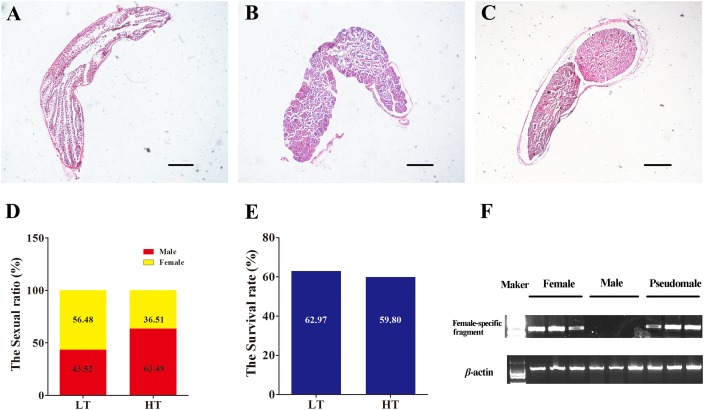
The identification of phenotype and genotype by and PCR. **(A–C)** Identification of phenotype, the gonadal histology of female, male and pseudomale. **(D)** The sexual ratio of LT and HT groups. **(E)** The survival rate of LT and HT group. **(F)** The identification of genotype by female-specific fragment.

The survival rates and proportion of females and males were counted both in LT group and HT group after treatment. No difference was found between the two groups in survival rate (χ^2^ = 0.190, *P* = 0.663). The survival rates were 62.97 and 59.80% in LT group and HT group (Figure 2E). The proportion of females and males as detected by biopsy and gonad tissue sectioning was 56.58 and 43.53% in LT group (Figure 2A–C), and 36.51 and 63.49% in HT group (Figure 2D), respectively. The proportion of males was significantly increased more than 20% after treatment with high temperature during TSP (χ^2^ = 7.624, *P* = 0.006). These data indicated that masculinization was induced in genetic females following high temperature treatment.

### RNA Sequencing

The genotypic and phenotypic sex of these individuals were distinguished by molecular marker and tissue section (Figure 2F). A total nine cDNA libraries were sequenced on the Illumina platform, generating 655,677,682 raw reads, encompassing about 30 Gb of sequence. Valid ratio and GC content of each cDNA library were shown in Table 1. Approximately 80.1% of reads exhibited significant hits to the genome. The transcriptome data obtained from the samples has been uploaded to NCBI SRA site, with accession numbers of PRJNA480118 (SAMN09628942, SAMN09628943, SAMN09628989, SAMN09628990, SAMN09628991, SAMN09628992, SAMN09628993, SAMN09628994, and SAMN09628995).

**Table 1:** Summary statistics of gonad transcriptome sequencing data.

Sample	Raw reads	Clean reads	Error	Q20	Q30	GC
Female-1-1	19757381	13615230	0.03	96.81	93.42	48.92
Female-1-2	19757381	13615230	0.03	95.56	91.42	48.92
Female-2-1	19500137	13249659	0.03	96.67	93.12	49.79
Female-2-2	19500137	13249659	0.03	95.83	91.88	49.79
Female-3-1	16918995	16322468	0.03	96.70	91.61	48.96
Female-3-2	16918995	16322468	0.03	96.72	91.61	48.95
Male-1-1	14492850	13853200	0.03	95.56	92.03	46.69
Male-1-2	14492850	13853200	0.03	95.61	92.00	46.78
Male-2-1	15806041	12193814	0.03	96.88	93.80	47.18
Male-2-2	15806041	12193814	0.03	95.73	91.97	47.14
Male-3-1	17961443	13498787	0.03	96.54	93.11	46.25
Male-3-2	17961443	13498787	0.03	95.45	91.30	46.33
Pseudomales-1-1	19970045	13033818	0.03	97.05	94.08	47.09
Pseudomales-1-2	19970045	13033818	0.03	95.40	91.43	46.98
Pseudomales-2-1	21633228	12794338	0.03	96.41	92.76	48.13
Pseudomales-2-2	21633228	12794338	0.04	94.95	90.50	48.02
Pseudomales-3-1	18792243	12507202	0.03	96.99	94.00	46.91
Pseudomales-3-2	18792243	12507202	0.03	94.98	90.76	46.80


### Differential Expression and Functional Enrichment Analysis

**FIGURE 3:**
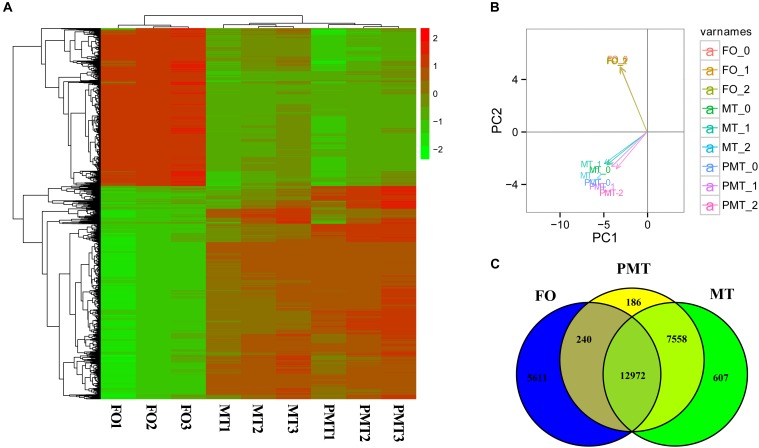
The expression profiles and DEGs among females, males and pseudomales in gonads. **(A)** Heatmap analysis of hierarchical clustering of DEGs in females (FO1, FO2, FO3), males (MT1, MT2, MT3), and pseudomales (PMT1, PMT2, PMT3). Each column represented an individual, and each row represented a gene. The FPKM was transformed by log10. Red color represented the high expressed genes, and green color represented the low expressed genes. **(B)** PCA plot of specimens. **(C)** Venn diagram shown the numbers of expressed genes and DEGs in FO, MT and PTM.

Principle component analysis analysis were conducted to detect the global similarities and differences expression profiles among FO, MT, and PMT. It displayed that ovary (FO) replicates clustered closely in a region, and testis (MT and PMT) replicates clustered into another region. The MT and PMT replicates clustered together (Figure 3B). These results demonstrated that the expression patterns of phenotypic females and males was significantly different. However, the expression profiles showed more similarity between males and pseudomales. Although females and pseudomales retained the same genotype, the expression profiles were quite different. Males and pseudomales possessed different sex chromosomes, but the expression patterns were similar.

Among the DEGs, 5851 genes were significantly differentially expressed in FO vs. MT. 5611 genes were found differentially expressed in FO vs. PMT. Between MT vs. PMT, only 426 genes were identified as DEGs (Figure 3C and Supplementary Table S1). Regarding the functions of the DEGs, a large number of genes related to gonad development and sex differentiation were identified, which include *Dmrt1, Dmrt3, HSD3b1, AMH, HSD3b7, esr1, SOX9, GATA4, GATA6, cyp19a, AMHR2* (Table 1). The heatmap of hierarchical clustering of DEGs was generated to visualize the expression patterns. The profile of phenotypic female was obviously different with all phenotypic male. The expression pattern of pseudomale was prone to that of male (Figure 3A).

**FIGURE 4:**
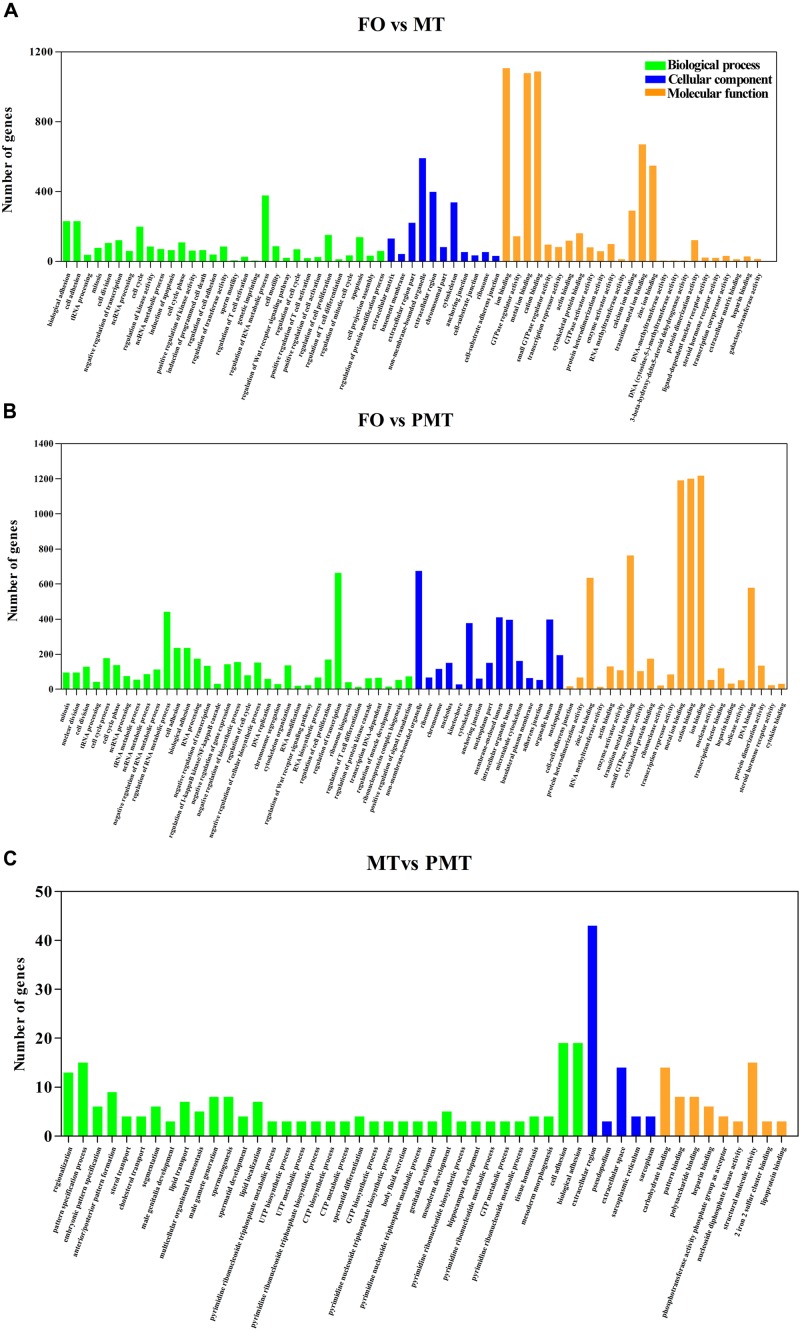
GO enrichment analysis of DEGs. DEGs were annotated to different GO terms in biological process, molecular function, and cellular component. **(A)** GO enrichment analysis of DEGs in FO vs MT. **(B)** GO enrichment analysis of DEGs in FO vs PMT. **(C)** GO enrichment analysis of DEGs in MT vs PMT.

**FIGURE 5:**
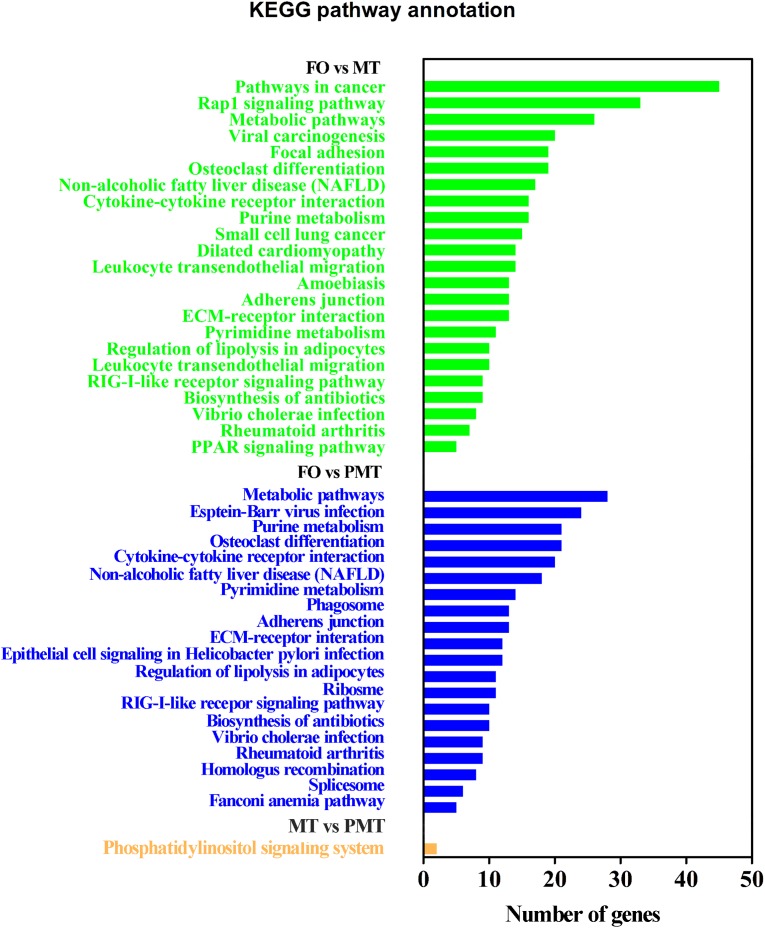
KEGG enrichment analysis of DEGs.

After filtration, the DEGs were applied to perform GO analysis and KEGG enrichment. All the DEGs were mapped to GO terms and compared with the background of the whole transcriptome. They were significantly enriched in several GO terms in biological process, cellular component and molecular function (Supplementary Table S2). The results of enrichment were as follow: (1) In DEGs of FO vs. MT, the terms related to sexual differentiation and the regulation of reproduction were enriched, including sperm motility, 3-beta-hydroxy-delta5-steroid dehydrogenase activity and steroid hormone receptor activity. Besides, the terms about immune response were enriched (Figure 4A). (2) In FO vs. PMT, the terms of steroid hormone and helicase activity were detected, such as steroid hormone receptor activity and helicase activity (Figure 4B). They were also involved in reproduction and sexual differentiation and development. (3) In MT vs. PMT, it was found that some terms related to reproduction and the generation and development of testis was detected, comprising of male gamete generation, spermatogenesis, spermatid development, spermatid differentiation, and sterol transport (Figure 4C). Interestingly, the terms about sperm generation and differentiation were detected, including male gamete generation, spermatogenesis and spermatid differentiation and development. Surprisingly, the GO terms about energy metabolism were enriched, including UTP metabolic process, CTP metabolic process, CTP biosynthetic process, GTP metabolic process (Figure 4C and Supplementary Table S2). These terms are involved in meiosis and gamete generation, and may influence sperm activity. Meanwhile, KEGG pathway enrichment analysis was performed. A total of 44 KEGG terms were significantly enriched. The enriched signal pathways were similar in FO vs. MT and FO vs. PMT, including ribosome biogenesis, cell adhesion and metabolism and biosynthesis. Only one signal pathway involved in metabolism was enriched, phosphatidylinositol signaling system (Figure 5).

### Identification of Genes Involved in Sexual Differentiation and Gonad Development

To identify genes involved in reproduction, including gonad development, gametogenesis and steroid biosynthesis in *C. semilaevis*, three strategies were used. (1) Sex-related genes were retrieved from the enriched GO terms, related to reproduction and steroid. (2) The DEGs were filtered by a set of key words that had been reported in other teleost, including gonad, sex, oocyte, meiosis, steroid, reproduction, and morphogenesis (Fan et al., 2014; Shao et al., 2014; Robledo et al., 2015). (3) Part genes were chosen from sex-related KEGG pathways. In accordance with these strategies, a set of potential candidate genes were obtained, and qRT-PCR validation were conducted (Table 2). Additionally, the DEGs of MT vs. PMT were analyzed independently, and the genes involved in spermatogenesis, gamete generation and development and energy metabolism were selected (Table 3).

**Table 2:** DEGs associated with sex differentiation and gonad development in FO vs. MT and FO vs. PMT.

Gene	FPKM		*P*-value	Annotation
			
	Female	Male		
**FO vs. MT**				
LOC103389072	0	4.79	5.00E-05	Spermatid perinuclear RNA-binding protein-like, partial
*spata22*	0	5.94	5.00E-05	Spermatogenesis-associated protein 22
*dmrt1*	0	62.29	5.00E-05	Doublesex- and mab-3-related transcription factor 1
*dmrt3*	0	42.99	5.00E-05	Doublesex- and mab-3-related transcription factor 3
*HSD3b1*	0.24	48.53	0.0001	3 beta-hydroxysteroid dehydrogenase
*AMH*	1.20	138.89	5.00E-05	Muellerian-inhibiting factor
LOC103389439	0.39	40.41	5.00E-05	Steroid 17-alpha-hydroxylase
LOC103384146	0.23	22.01	5.00E-05	EGF-like module-containing mucin-like hormone receptor-like 1
LOC103381541	0.37	30.15	5.00E-05	Lutropin-choriogonadotropic hormone receptor-like isoform X1
*HSD3b7*	0.25	18.96	0.0001	3 beta-hydroxysteroid dehydrogenase type 7
LOC103388483	1.06	75.06	5.00E-05	Steroid 21-hydroxylase isoform X1
*spef2*	0.06	2.98	0.0001	Sperm flagellar protein 2
LOC103381193	2.35	89.68	5.00E-05	Estrogen receptor beta-like isoform X1
*esr1*	0.22	6.48	5.00E-05	Estrogen receptor isoform X2
*Sox9*	0.36	10.15	5.00E-05	Transcription factor SOX-9
*amhr2*	2.14	51.65	5.00E-05	Anti-Muellerian hormone type-2 receptor
*GATA6*	1.59	35.29	5.00E-05	Transcription factor GATA-6
*topaz1*	0.71	14.88	5.00E-05	Testis- and ovary-specific PAZ domain-containing protein 1
*GATA4*	0.54	10.33	5.00E-05	Transcription factor GATA-4
*srd5a2*	1.67	26.28	5.00E-05	3-oxo-5-alpha-steroid 4-dehydrogenase 2
*ddx17*	3.22	26.45	5.00E-05	Probable ATP-dependent RNA helicase DDX17
*HSD17b1*	3.42	13.93	5.00E-05	Estradiol 17-beta-dehydrogenase 1
LOC103386902	2.55	10.32	5.00E-05	Oocyte zinc finger protein XlCOF6-like
*spata5l1*	45.09	8.41	5.00E-05	Spermatogenesis-associated protein 5-like protein 1
*ebp*	75.74	8.35	5.00E-05	3-beta-hydroxysteroid-Delta(8), Delta(7)-isomerase
LOC103377895	37.95	4.18	5.00E-05	Oocyte zinc finger protein XlCOF6-like
*cyp19a1a*	5.85	2.14	5.00E-05	Aromatase-like
**FO vs. PMT**				
LOC103388599	0	5.70	5.00E-05	R-spondin-3-like
LOC103389072	0	4.06	5.00E-05	Spermatid perinuclear RNA-binding protein-like, partial
*spata22*	0	3.07	5.00E-05	Spermatogenesis-associated protein 22
*dmrt1*	0	55.04	5.00E-05	Doublesex- and mab-3-related transcription factor 1
*dmrt3*	0	64.61	5.00E-05	Doublesex- and mab-3-related transcription factor 3
*HSD3b1*	0.24	51.35	0.0001	3 beta-hydroxysteroid dehydrogenase
*AHM*	1.20	146.5	5.00E-05	Muellerian-inhibiting factor
LOC103388483	1.06	121.78	5.00E-05	Steroid 21-hydroxylase isoform X1
*HSD3b7*	0.25	25.85	5.00E-05	3 beta-hydroxysteroid dehydrogenase type 7
LOC103389439	0.39	36.53	5.00E-05	Steroid 17-alpha-hydroxylase/17,20 lyase-like
LOC103381193	2.35	110.53	5.00E-05	Estrogen receptor beta-like isoform X1
*esr1*	0.22	9.96	5.00E-05	Estrogen receptor isoform X2
*amhr2*	2.14	93.25	5.00E-05	Anti-muellerian hormone type-2 receptor
*Sox9*	0.36	8.77	5.00E-05	Transcription factor SOX-9
*smox*	5.35	41.32	5.00E-05	Spermine oxidase isoform X2
LOC103387725	3.38	20.17	5.00E-05	Steroid 17-alpha-hydroxylase/17,20 lyase
*meiob*	123.82	8.06	5.00E-05	Meiosis-specific with OB domain-containing protein isoform X2


**Table 3:** DEGs associated with spermatogenesis and energy metabolism in MT vs. PMT.

Gene	FPKM		*P*-value	Annotation
			
	Male	Pseudomale		
**Spermatogenesis**				
LOC103396997	0.56	2.67	5.00E-05	Endothelial lipase-like
LOC103394959	1.33	8.987	5.00E-05	ATP-binding cassette sub-family G member 4-like
*ldlr*	0.53	8.227	5.00E-05	Low-density lipoprotein receptor
*spef1*	13.57	57.88	5.00E-05	Sperm flagellar protein 1
LOC103380361	11.79	48.08	5.00E-05	LOW QUALITY PROTEIN: oxysterol-binding protein-related protein 5-like
LOC103394710	2.60	17.04	5.00E-05	Kelch-like protein 10 isoform X3
*tbx3*	2.77	13.81	5.00E-05	T-box transcription factor TBX3 isoform X2
LOC103386655	3.39	19.34	5.00E-05	Kelch-like protein 10
LOC103380607	0.311	1.75	5.00E-05	MYCBP-associated protein-like
LOC103390132	2.16	9.965	5.00E-05	Galactosylceramide sulfotransferase-like
LOC103398025	4.40	0.685	5.00E-05	Spermatid perinuclear RNA-binding protein-like isoform X2
LOC103391171	1.73	19.68	5.00E-05	Nucleoside diphosphate kinase homolog 5-like
LOC103397033	0.41	1.895	5.00E-05	Citron Rho-interacting kinase-like, partial
LOC103395317	6.11	43.27	5.00E-05	DNAJ homolog subfamily B member 13-like isoform X2
**Energy metabolism**				
LOC103391171	1.73	19.68	5.00E-05	Nucleoside diphosphate kinase homolog 5-like
LOC103393462	3.14	50.06	5.00E-05	Nucleoside diphosphate kinase A-like
LOC103392972	7.17	29. 70	5.00E-05	Nucleoside diphosphate kinase, mitochondrial-like isoform X2


### qRT-PCR Validation

**FIGURE 6:**
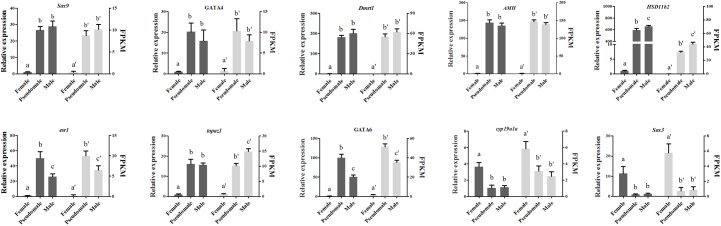
Verification of the expression patterns both in qRT-PCR and RNA-seq. The data was shown as mean ± SD (*n* = 6). Groups with different letters were significantly different (*P* < 0.05).

The expression patterns of ten DEGs (*Sox9, GATA4, Dmrt1, AMH, HSD11b2, cyp19a1a, esr1, topaz1, GATA6, Sox3*) associated with gonad development or steroid biosynthesis were selected for qRT-PCR validation. All the genes displayed consistent expression patterns both in qRT-PCR and RNA-seq (Figure 6). The Pearson correlation coefficient analysis exhibited correlation between qRT-PCR assay and RNA-seq data (*R* = 0.394, *P* = 0.031), indicating the accuracy and reliability of RNA-seq.

### Sex-Specific Methylation Levels of Gonadal *cyp19a1a* Promoter

The DEGs analysis, qRT-PCR validation as well as the results of previous study (Shao et al., 2014), indicated that *cyp19a1a* played an essential role in sex differentiation, and sex reversal induced by temperature in *C. semilaevis*. It was regarded that proper expression of *cyp19a1a* is essential for maintaining the ratio of androgen and estrogen. The balance might be destroyed by expression changes of *cyp19a1a* mediated by abnormal environmental temperature. Epigenetic modification is considered as one of the factors that might affect *cyp19a1a* expression level.

**FIGURE 7:**
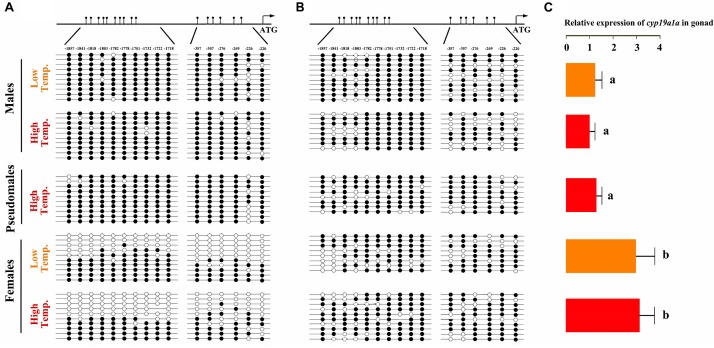
*C. semilaevis cyp19a1a* promoter methylation levels and correlation with gonad gene expression according to sex and temperature treatment. **(A)** The methylation patterns of *C. semilaevis cyp19a1a* promoter in gonads. **(B)** The methylation patterns of *C. semilaevis cyp19a1a* promoter in muscle. Numbers indicated CpG positions with respect to the transcription starting site. Open and filled circles denoted unmethylated or methylated positions, respectively, while no circles denoted unknown methylation status due to sequencing problems. Eight to ten clones per fish were analyzed. **(C)** The relative expression level of *C. semilaevis cyp19a1a* in gonads by qRT-PCR. The data was shown as mean ± SD (*n* = 6). Groups with different letters were significantly different (*P* < 0.05).

To test our hypothesis, DNA methylation of *cyp19a1a* promoter in gonad and muscle was examined. The CpG dinucleotides ∼2000 bp upstream of the transcription start site were selected, which had two approximate clusters: 10 CpGs in the distal promoter region (-1857 to -1718, designated as region I) and 6 CpGs in the proximal promoter region (-357 to -220, designated as region II). No difference in the methylation level was detected in the muscle tissue among females, males and pseudomales (Figure 7B). In the gonads, however, significant higher methylation levels were observed in male and pseudomale testis than in the female ovaries (Figure 7A). It was important to notice that high temperature-induced sex reversal from females to pseudomales is accompanied by the significant elevation of methylation level of gonadal *cyp19a1a* promoter.

To investigate if the promoter methylation would regulate the expression of *cyp19a1a*, qRT-PCR was performed in gonads of three groups. The expression level in females from LT and HT groups was similar, which were significantly higher (*P* < 0.05) than that in males (both LT and HT groups) as well as pseudomales, No expressional difference was observed between males and pseudomales (*P* > 0.05) (Figure 7C). Based on the methylation and expression data, we conclude that the expression level of *cyp19ala* showed highly negative correlation with the promoter methylation levels in gonads.

However, it was not the same case in the muscle tissue, where the *cyp19a1a* was only basally expressed. The average methylation levels of *cyp19a1a* promoter were similar and high in all, regardless of temperature treatment (Figure 7B). Two-way ANOVA analysis showed absolutely no differences among three groups in the *cyp19a1a* promoter methylation level in terms of temperature treatment (*P* > 0.05) and sex (*P* > 0.05). Either, no significant interaction between the two factors was found (*P* > 0.05).

### DNA Methylation Inhibits cAMP-Stimulated *cyp19a1a* Promoter Activity *in vitro*

**FIGURE 8:**
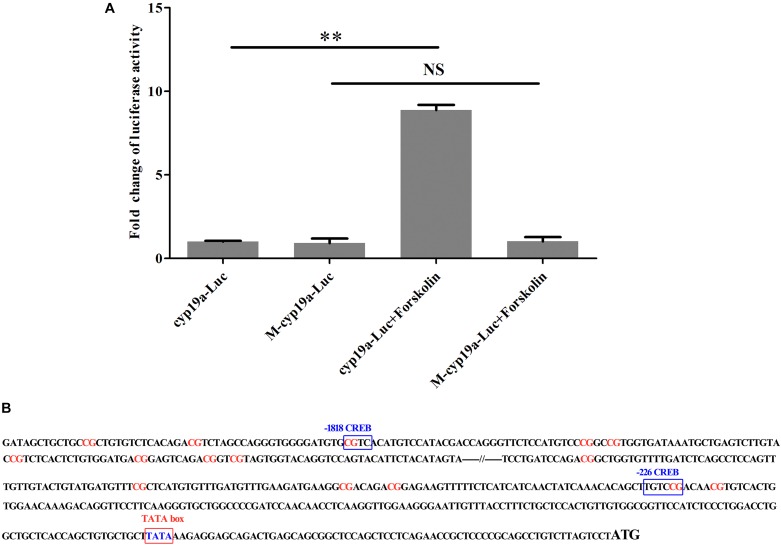
The effect of DNA methylation of *cyp19a1a* promoter on cAMP-stimulated activities. Unmethylated and methylated reporter plasmids were transfected to HEK 293T cells. Luciferase activities were measured 48 h after transfection. Fold change was calculated, and *cyp19a1a*-Luc group was used as control. **(A)** The CREB mediated stimulation of *cyp19a1a* promoter activities by forkolin in HEK 293T cells. **(B)** The location of CG sites and CREB sites in two approximate clusters of *cyp19a1a* promoter. The data was shown as mean ± SD (*n* = 3). ^∗∗^*P* < 0.01 represented significantly different.

Transcription factor binding sites in *cyp19a1a* promoter were predicted using MatInspector. Two binding sites for CREB were found in the CpGs in position -1818 and -226, respectively (Figure 8B). *In vitro* study demonstrated that the methylation could decrease the activity of *cyp19a1a* promoter. The activity of unmethylated promoter could be significantly induced by forskolin stimulation. In contrast, no significantly change was observed in methylated promoter (Figure 8A).

## Discussion

Since the initial discovery of vertebrate GSD + TE, the mechanism by which temperature exerts its influence on sex determination has been extensively investigated (Ferguson and Joanen, 1982; Rhen and Schroeder, 2010; Czerwinski et al., 2016; Schroeder et al., 2016; Yatsu et al., 2016). Sex reversal can be induced when the temperature achieved a threshold, and cause sex ratio change. The sex of embryos, larva or juveniles can be reversed completely or partly under a threshold temperature in reptiles and teleost (Ferguson and Joanen, 1982; Strüssmann et al., 1996; Hattori et al., 2013; Czerwinski et al., 2016). The pseudomales have the same chromosome complement with females, but the phenotype is completely different (Hu et al., 2014). Usually, temperature exerts its influence at TSP of embryo, larva or juvenile development, when the individuals remain sexually flexible (Navarro-Martín et al., 2011; Holleley et al., 2015).

In this study, a teleost, *C. semilaevis*, sensitive to temperature was used. We demonstrated the significant imbalance of sex ratio and survival rate after high temperature treatment in TSP. The proportion of males was about 20% higher in HT group, indicating masculinization was induced by high temperature. This phenomenon have also been reported in *M. menidia, D. labrax, O. niloticus*, and *O. mykiss* (Conover and Kynard, 1981; Navarro-Martín et al., 2011; Valdivia et al., 2014; Wang et al., 2017). Interestingly, only part of the females were easy to be induced sex reversal. In a recent study, a SNP (A/T) of *FBXL17* had large controlling effect on sex reversal in *C. semilaevis*, and Z^A^W genotype would never reverse into phenotypic males, while those with Z^T^W genotypes would sometimes undergo sex reversal (Jiang and Li, 2017). Based on these results, we speculated that some mutation might cause the females to be sensitive to temperature, and sex reversal emerged when the temperature exceeded threshold.

To explore the different expression profiling of female, male and pseudomale, RNA-seq was performed. A lot of GO terms involved in reproduction and steroid biosynthesis were identified by DEGs and enrichment analysis. Interestingly, some GO terms related to immune responses were also enriched. Similar results were displayed in *Pogona vitticeps*, in which the expression levels of prominent immune genes were significantly lower in pseudomales than in females and males. Further, canonical stress-related GO terms were enriched, including defense response, response to biotic stimuli (Deveson et al., 2017). It has been known that immune system was intertwined with stress. Meanwhile, evidences showed that stress and sex were connected in vertebrates. In *Amphiprion akallopisos* and *Odontesthes bonariensis*, cortisol was considered the regulator of sex change in response to environmental or social stress (Hattori et al., 2009; Yoshinaga et al., 2010; Fernandino et al., 2012, 2013; Kitano et al., 2012; Todd et al., 2016). In reptiles, POMC and corticosterone-mediated stress was observed in sex-reversed individuals (Deveson et al., 2017). In birds and rats, elevated maternal corticosterone and ACTH skewed the sex ratio of offspring (Barbazanges et al., 1996; Pike and Petrie, 2006). In human, evidence indicated that maternal stress could enhance the circulation of corticosterone and affect neuroendocrine system. These stresses had long-lasting effects on offspring morphology, behavior, physiology, and phenotype, which could cause the imbalance of sex ratio (Obel et al., 2007; Navara, 2010; Schnettler and Klüsener, 2014; van den Heuvel et al., 2018). According to a series of studies, we speculated that the *C. semilaevis* larva was stressed by high temperature, and immune response was activated. Then, these responses influenced endocrine system, which caused the up-regulation or down-regulation of cortisol. The biosynthesis and secretion of steroid were interfered, which leaded to sex reversal under the stress of high temperature treatment. Till now, the evidence has not been adequate, so the interaction of stress and endocrine and specific mechanism need further study.

A series of evidences of environmental influences on phenotype plasticity in vertebrate mediated by epigenetic mechanisms, such as DNA methylation and histone deacetylation has been obtained (Reik et al., 2001; Jaenisch and Bird, 2003). Epigenetic regulation can inhibit or stimulate gene transcription, which alters gene expression from the same genetic blueprint and thus affects development and differentiation (Rottach et al., 2009). In previous studies, whole-genome methylation has been found to be involved in sex-induced by high temperature in *C. semilaevis*, and methylation modification in sex-reversed males was inherited. Besides, dosage of Z chromosomal region was related to sex reversal in *C. semilaevis* (Chen et al., 2014; Shao et al., 2014). However, it was found higher levels of methylation of *cyp19a1a* and also higher levels in gene expression of *cyp19a1a* (Shao et al., 2014). In the present study, we found that the methylation level of *C. semilaevis cyp19a1a* promoter was significantly higher in males than in females. Importantly, the methylation profiles of pseudomales were similar with males, but absolutely different from females, although pseudomales had the same genotype (ZW) with females. Based on the methylation and expression data, we concluded that the expression level of *cyp19a1a* showed highly negative correlation with the promoter methylation levels in ovaries and testes. In *Oryzias latipes*, DNA methylation of *cyp19a1a* promoter was reported to be related to sex differentiation (Contractor et al., 2004). The methylation levels were twice in males compared with females in *D. labrax* gonads (Navarro-Martín et al., 2011). Besides, the allied discoveries was also observed in *O. niloticus* and *P. olivaceus* (Fan et al., 2017; Wang et al., 2017). *Cyp19a1a* played important roles in sex differentiation by regulating estrogen synthesis. In *C. semilaevis*, females and pseudomales had the same genetic background (ZW), but different DNA methylation and expression levels of *cyp19ala*. Epigenetic modification caused by high temperature might transform the topology of DNA and block the binding of transcription factor, which could change the expression of *cyp19a1a*. *In vitro* study demonstrated that the methylation of -1818 and -226 sites in *cyp19a1a* promoter inhibited the binding of transcription factor CREB and suppressed the promoter activity, which could regulate the expression level of *cyp19a1a*. Thus, our results clearly showed that epigenetic modification, most likely DNA methylation, regulated the expression of gonadal *cyp19a1a*, which then mediated sex differentiation.

**FIGURE 9:**
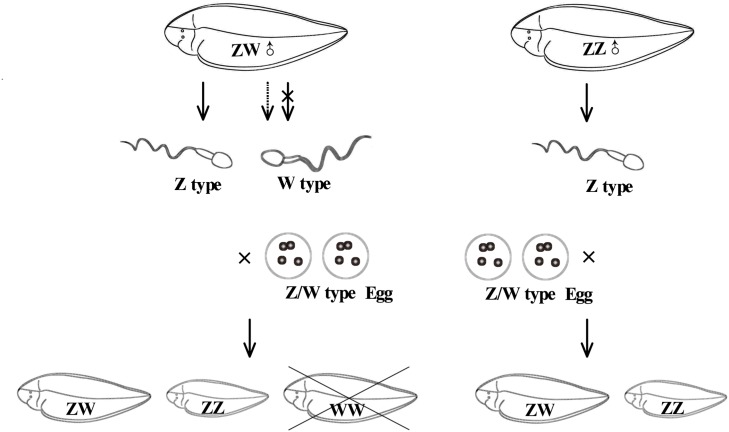
The possible diagram of generating sperm and reproducing next generation in pesudomales and normal males.

Interestingly, a lot of DEGs between males and pseudomales were enriched to GO terms involved in spermatogenesis, including spermatogenesis, male genitalia development, male gamete generation, spermatid development, and spermatid differentiation. Both males and pseudomales generate sperms, but the process seemed to be significantly different. In males, only Z type sperms were generated, but both Z type and W type sperms were generated theoretically in pseudomales. The generation of different types of sperm might influence spermatogenesis and spermatid differentiation and development. Surprisingly, GO terms related to energy metabolism such as UTP, GTP, and CTP biosynthetic process and metabolic process were also enriched in DEGs between males and pseudomales. Energy metabolism could affect the sperm vitality. The results implied the quality of sperm generated from males and pseudomales might be significantly different. In theory, super female (WW) individuals could be generated by W type sperm fertilized with W type eggs. However, super females were never observed in the larval stage in our lab produced by pseudomales (unpublished data). These lines of evidence suggest that W type sperm generated from pseudomale might have weak vitality. Pseudomales might unable to generate function W type sperms or the WW embryos could not develop normally to larva (Figure 9).

## Conclusion

In conclusion, we demonstrated that high temperature could induce masculinization in *C. semilaevis*. The expression patterns of pseudomales was similar to males, but the genes involved in spermatogenesis and energy metabolism were differentially expressed. Besides, high-temperature treatment could change the epigenetic modification of *cyp19a1a* promoter, leading to DNA methylation level increase in pseudomales, which results in the decrease of *cyp19a1a* expression. There was a negative correlation between methylation levels and expression of *cyp19ala*. Thus the epigenetic regulation of *cyp19a1a* might play an essential role in the sex reversal induced by high temperature in *C. semilaevis*.

## Ethics Statement

This study was carried out in accordance with the recommendations of the Administration of Affairs Concerning Experimental Animals. The protocol was approved by the College of Marine Life, Ocean University of China.

## Author Contributions

JL, XL, CJ, and XD performed the experiments and analyzed the data. JL and YH prepared the figures and wrote the manuscript. QZ designed the experiments.

## Conflict of Interest Statement

The authors declare that the research was conducted in the absence of any commercial or financial relationships that could be construed as a potential conflict of interest.

## Supplementary Material

TABLE S1 DEGs in FO vs. MT, FO vs. PMT, and MT vs. PMT.

TABLE S2 GO terms in biological process, cellular component, and molecular function levels.

TABLE S3 List of primer sequences used in the study.
