# Supplementary material for: Transcriptome Profiling Insights the Feature of Sex Reversal Induced by High Temperature in Tongue Sole Cynoglossus semilaevis
Source: Front Genet. 2019 May 29;10:522. doi: 10.3389/fgene.2019.00522 (PMC6548826; doi:10.3389/fgene.2019.00522)
Supplement: TABLE S3 — List of primer sequences used in the study. [file Table_3.docx]

Table 3 List of primer sequences used in the study.

| Primers | Sequence (5′-3′) | Tm (°C) | Usage |
| --- | --- | --- | --- |
| Female-specific-Fw  Female-specific-Rv  Sox9-Fw | AATGCTTTCTTTAGCGTTCT  GTGTTTCTTTGTGATGGGTT  GAGGCAGAGAGAAAGAGAGA | 58  58  60 | sex certification  sex certification  qRT-PCR |
| Sox9-Rv | CACGTGAATCGAAGGATGAA | 60 | qRT-PCR |
| GATA4-Fw | CACACACAGATCTCAGCCTTAC | 60 | qRT-PCR |
| GATA4-Rv | GGCTTTGGAGCTGGAAGTTA | 60 | qRT-PCR |
| Dmrt1-Fw | GTCGCTGTGACAAGTGTAACCTC | 60 | qRT-PCR |
| Dmrt1-Rv | TGAGACATCTGCTGGTATTGCTG | 60 | qRT-PCR |
| AMH-Fw | ATGTGGCTGTAGAAGAAGAC | 60 | qRT-PCR |
| AMH-Rv | CTGTTAGCAGGATGTATCG | 60 | qRT-PCR |
| HSD11b2-Fw | CACTCCGGTGACTCAAATAG | 60 | qRT-PCR |
| HSD11b2-Rv | AGGAGGTGAGACCAAAGT | 60 | qRT-PCR |
| cyp19a1a-Fw | ACCTCAGCATGACTTTCTTAC | 60 | qRT-PCR |
| cyp19a1a-Rv  esr1-Fw  esr1-Rv  topaz1-Fw  topaz1-Rv  GATA6-Fw  GATA6-Rv  Sox3-Fw  Sox3-Rv | CTGGTGTAACCCAACCTTATT  TACAGGACATGCTGGAAATG  GTGTCTGATGTGTGAGAGAAG  TATGGAGTTTAACAGGCGATT  GTCCGCAAAGTTTACAACAG  CGAGTCCCTACTCTCCTTATGT  CTCTGTAGAGTGTGCAGCATAG  CGAGAGAGAGAGAGACTAGAAG  CACGACTCACAGAGGTAGA | 60  60  60  60  60  60  60  60  60 | qRT-PCR  qRT-PCR  qRT-PCR  qRT-PCR  qRT-PCR  qRT-PCR  qRT-PCR  qRT-PCR  qRT-PCR |
| β-actin-Fw | GCTGTGCTGTCCCTGTA | 60 | qRT-PCR |
| β-actin-Rv | GAGTAGCCACGCTCTGTC | 60 | qRT-PCR |
| GAPDH-Fw | GAAGGGCATTCTGGGATACACT | 62 | qRT-PCR |
| GAPDH-Rv  M-cyp19a1a-Fw1  M-cyp19a1a-Rv1  M-cyp19a1a-Fw2  M-cyp19a1a-Fw1  pGL3-cyp19a1a-Fw  pGL3-cyp19a1a-Rv | TCAAAGATGGAGGAGCGGC  TTTATATTTATTTTGATAGTTGTTGT  CTATATAAAATATACTAAACCTATACCACT  ATGGGAAAAATGTTTGAAATTTTAA  ACCAACACCCTTAAAAAAACCTATC  CGGGGTACCCCACACCCACTCTGATAGCTG  CCGCTCGAGCTATTAGCGTCTCCTCTCCATTG | 62  58  58  55  55  56  56 | qRT-PCR  BSP-PCR  BSP-PCR  BSP-PCR  BSP-PCR  Plasmid construction  Plasmid construction |
